# Environmentally Responsive Materials for Building Envelopes: A Review on Manufacturing and Biomimicry-Based Approaches

**DOI:** 10.3390/biomimetics8010052

**Published:** 2023-01-26

**Authors:** Maria De Los Ángeles Ortega Del Rosario, Kimberly Beermann, Miguel Chen Austin

**Affiliations:** 1 Faculty of Mechanical Engineering, Universidad Tecnológica de Panamá, Panama City 0819, Panama; 2 Sistema Nacional de Investigación (SNI), Clayton City of Knowledge Edf. 205, Panama City 0819, Panama; 3 Geography Department, Birkbeck, University of London, London WC1E 6BT, UK; 4 International Association for Hydro-Environment Engineering and Research (IAHR), Panama Young Professionals Network (YPN), Panama City 0801, Panama; 5 Centro de Estudios Multidisciplinarios en Ciencias, Ingeniería y Tecnología (CEMCIT-AIP), Panama City 0819, Panama

**Keywords:** biomimetic, biomimicry, environmental, natural inspiration, manufacturing, responsive materials, responsive building envelopes

## Abstract

Buildings must adapt and respond dynamically to their environment to reduce their energy loads and mitigate environmental impacts. Several approaches have addressed responsive behavior in buildings, such as adaptive and biomimetic envelopes. However, biomimetic approaches lack sustainability consideration, as conducted in biomimicry approaches. This study provides a comprehensive review of biomimicry approaches to develop responsive envelopes, aiming to understand the connection between material selection and manufacturing. This review of the last five years of building construction and architecture-related studies consisted of a two-phase search query, including keywords that answered three research questions relating to the biomimicry and biomimetic-based building envelopes and their materials and manufacturing and excluding other non-related industrial sectors. The first phase focused on understanding biomimicry approaches implemented in building envelopes by reviewing the mechanisms, species, functions, strategies, materials, and morphology. The second concerned the case studies relating to biomimicry approaches and envelopes. Results highlighted that most of the existing responsive envelope characteristics are achievable with complex materials requiring manufacturing processes with no environmentally friendly techniques. Additive and controlled subtractive manufacturing processes may improve sustainability, but there is still some challenge to developing materials that fully adapt to large-scale and sustainability needs, leaving a significant gap in this field.

## 1. Introduction

The global outlook on material and energy usage, as well as water footprint, pollution, environmental impacts, climate change, and health issues, has led most productive sectors to analyze their processes and products exhaustively. These efforts aim to determine the actions to mitigate their adverse effects. A sector of great interest due to the impact of these actions is the building construction sector. In this sense, the construction sector is currently responsible for 40% of solids generation, 36% of global energy demand, 12% of drinking water depletion, 37% of energy-related CO_2_ emissions, and 38% of greenhouse gas emissions [1]. However, concrete manufacturing is responsible for 8% of anthropogenic CO_2_ emissions [2]. Although, even though the COVID-19 pandemic temporarily reduced the energy demand, lowering the contribution of CO_2_ emissions in this sector by an estimated 10% back in 2020 [1], these key figures show the prominent role of this sector.

In addition, construction also plays a key role as an economic sector. Until 2019, it represented 6% of the world’s gross domestic product (GDP), and it is projected to reach 14.7% by 2030 [3]. With the COVID-19 pandemic, the development of the building and construction sectors is instrumental in recovery and stimulus plans, offering a new pathway [1] by improving their environmental and health sustainability [4,5,6,7].

Furthermore, cities and building development, as well as their residents, play a significant role in achieving sustainability. This states challenges in reaching goals related to the neutrality of emission, material use, water, and other non-renewable sources for their construction and maintenance [8,9]. Population growth has increased cities’ expansion and the need for construction [10]. These challenges make the design, architecture, performance, and life-cycle assessment of buildings and cities a significant research trend [11].

Buildings must adapt and respond dynamically to their environment to reduce their energy loads and mitigate environmental impacts [12,13]. Adaptive and regenerative buildings can tackle building energy consumption and carbon dioxide emissions, benefiting in terms of comfort and mitigation of the heat island effects [14,15,16,17], which changes the perspective of the envelope traditional static solution [17]. In this sense, responsive building envelopes have shown potential for energy savings, reducing energy inefficiencies and greenhouse gas emissions and improving heating, cooling, and light uses [16]. The envelope plays an essential role in regulating the exchanges between the indoors and the outdoors of a building [17]. It contributes significantly to the energy-saving potential and low environmental impact of a building [18] while providing comfort for the occupants [19].

According to Romano et al. [20], and resumed by Borkowski et al. [21], the characterization parameters of adaptive envelopes and facades include at least one of the following: high-performance, innovative materials, and systems to absorb and store solar energy; devices to manage natural and mechanical ventilation systems; mobile screens to control solar radiation; technological solutions to enhance or control the comfort in the building; and building management systems (BMS) to manage plants and building envelope elements. All these pose the challenge of shifting buildings’ traditional design and architecture into a more sustainable, adaptative, and conscious approach that copes with the current needs [14,15]. However, material selection may also be challenging since finding materials suitable to the building sector depend on availability, climatic conditions, and costs [22].

Several approaches have been tackled to address responsive behavior in buildings, such as adaptive and biomimetic envelopes [17]. The latter has gained attention in the last years, in which nature is used as a source of inspiration through biomimetics or biomimicry approaches. According to Benyus [23], one of the precursors of the recent development of biomimicry, it can be defined as the science that studies nature’s models and then imitates or takes inspiration from these designs and processes to solve human problems. The author stated that this inspiration from nature could be executed by using nature as a model (organizational level), nature as a measure (behavior level), and nature as a mentor (system level). Several authors have studied and developed suitable frameworks to integrate biomimicry approaches to design in architecture. These approaches can be a solution-based approach (bottom-up) and a problem-based approach (top-down) [24,25,26]. The former approaches are categorized by biological and technological domains, while the latter first state the problem and then look into nature for solutions.

Biomimicry as a tool in architecture and building construction offers the opportunity to inspire active envelopes and integrate natural concepts and principles aiming for sustainable and climate responses [2,19,27,28,29]. According to Somesse et al. [17], biomimicry approaches can be taken advantage of in the architectural context using morphological–structural emulation, mimicking the morphology of a living being found in nature to recreate a specific behavior or response, and dynamic–functional emulation.

One major challenge of using biomimicry is that, in many cases, solutions are accompanied by complex geometries, leading to difficulties during material selection and manufacturing. Most of the structures achieved by nature result from a growth process, which may be challenging to replicate with a fabrication process, even more when one relies on traditional manufacturing techniques. During growth, form and microstructure are created in the same process; thus, for biological materials, the structure becomes hierarchical [30,31]. However, the latest advances in material design and development and the industrial development of manufacturing techniques within the industry 4.0 revolution, such as those integrating robotics and computer numerically controlled (CNC) [32], may ease these challenges.

Novel techniques in the building and construction sector, such as additive manufacturing (AM), have opened the door to achieving such complex geometries. AM shows several advantages since it allows for generating products with complex shapes, geometric freedom, and a high degree of detail [33], reducing human intervention and potential risks [34]. Therefore, AM has the potential to reduce the waste of materials, the labor time, and the time associated with the manufacturing process, improving sustainability in construction [35]. 

The development during the 1990s of computer-aided design (CAD), computer-aided modeling (CAM), and computer-aided engineering (CAE) have allowed engineers, designers, and architects to develop complex geometries [36] and product development tools, allowing engineers to model such products’ design responses and service performance [37]. However, despite the construction sector’s economic, social, and environmental impact, their fabrication methods are predominantly low-tech based on artisanal approaches, characterized by poor performance and quality [38], which results in a challenge for environmental adaptation of the building construction sector. According to the Industrial Digitization Index of the McKinsey Global Institute, the construction sector is the second least digitized in the US and the least digitized in the European Union [39]. As a result, the sector’s digitalization searches have intensified in the last decade, achieving significant advances regarding Building Information Modeling (BIM) [40]. However, the necessary efforts to achieve sustainability within the sector must be further intensified, adapting to new technologies.

All these technologies, together with others, such as BIM, may allow the building sector to rethink its products and lead architects, engineers, and designers can move toward disruptive approaches, including design thinking, biomimicry, and additive manufacturing [41]. Alternatives such as prefabrication may furthermore lead to effective envelope production [42]. Although, according to Attia et al. [18], some challenges in this integration of active envelopes that may face the building construction sector are: the need to adapt the current technology to operate adaptive facades optimally and the development of a proper performance assessment framework.

This study seeks to provide a critical and comprehensive review of biomimicry approaches to develop responsive envelopes, aiming to understand the connection between material and manufacturing processes with physical phenomena. A literature search-based methodology was used to understand the role of materials and the manufacturing processes in mimicking nature, as well as the challenges that achieving this may face. This article is organized into three sections: (1) biomimicry in responsive envelopes and applications, (2) biomimicry-based materials, and (3) biomimicry-based manufacturing processes. In the first section, the literature of recent studies using biomimicry to model or install active and responsive facades is critically reviewed with the aim of understanding how these approaches have been tackled in the past and, most importantly, how the authors have faced challenges in material design or adaptation and manufacturing techniques. Following this, section two reviews the use of materials to comply with biomimetics-based approaches in casing development. Section three encloses manufacturing techniques that have been adapted to achieve responsive envelopes or biomimicry-based approaches that may enhance manufacturing techniques. At the end of both sections, a discussion addresses possibilities, challenges, and the future of biomimicry-based materials and manufacturing techniques.

## 2. Materials and Methods

The recent use of biomimicry approaches to develop responsive envelopes has been previously addressed in critical reviews [2,17,24,25,27,28,29,31,43,44,45]. These authors provide a starting point to develop a general overview of the global context and development of responsive building envelopes using biomimicry. Among these works, one can find an understanding of the evolution of biomimicry approaches; responsive, reactive, adaptive, biomimetic, or kinetic envelopes; environmental factors that impact the envelope and the occupants; role models found in nature to mimic a behavior or function, a morphology or a movement; biomimicry materials and manufacturing processes; and biomimicry approaches to achieve these facades. However, even if there is a piece of broad information reviewed, there is still some room to improve the understanding of materials and manufacturing processes that allow tackling the physical implementation of reactive envelopes efficiently, depending on the environmental factor stimulating the envelope or the biomimicry solution proposed.

The first step of this methodology was to develop an initial literature search based on the global context of responsive envelopes and architecture and building development. This part aimed to understand the global outlook, research trends, and main subject of interest in the development of responsive building envelopes by using biomimicry approaches. In this section, a simple query of “biomimicry OR biomimetic.” From here, three research questions were posed.

- R.Q.1. What have been the biomimicry solutions previously adapted to responsive envelopes or related and their mechanisms?
- R.Q.2. How have materials been previously designed or adapted to comply with biomimicry solutions adapted to responsive envelopes?
- R.Q.3. Which manufacturing techniques have been used to comply with biomimicry solutions, or how has biomimicry enhanced manufacturing techniques adapted to responsive envelopes?

As in any product, the building responsive envelope performance depends heavily on the materials selected, which are inherently related to the processing and manufacturing to obtain the final product. Thus, the first question intended to provide an understanding of biomimicry approaches in building envelopes. This research question cements this study since materials and manufacturing cannot be selected appropriately without a deep understanding of the physical phenomena and the final application in which the product will perform. The second and third questions were on the role of materials and the manufacturing process in achieving an adequate response on using biomimicry as a source of inspiration to develop responsive facades.

A two-phase search was proposed for this literature review. The first phase included a search query that aimed to review the (i) mechanisms, (ii) species, (iii) functions or strategies, (iv) materials, (v) morphology, and their (vi) biomimicry approaches previously performed. For this, a simple query of title, keywords, and abstract was proposed as “biomimicry OR biomimetic” AND “…”, where the “…” represents each of the aiming concepts proposed before. This section was developed as an iterative process, in which by analyzing the title, abstract, and keywords proposed in the resulting articles, it was possible to identify the trends, key factors, and main issues related to the use of biomimicry in inspiring responsive building envelopes.

The second phase sought to land on the case studies relating to biomimicry approaches and envelopes. During this iterative process, it was noted that many of the developments of responsive building envelopes have been on facades. Therefore, it was included as a search word. Thus, facade OR envelope was added to the search query as (biomimicry OR biomimetic) AND (facade OR envelope) AND “…”.

Scopus and Web of Science were used as scientific databases, with inclusion criteria of articles, book chapters, conference papers, technical notes, and review papers of the last five years (2018–actuality) for building construction and architecture-related studies. This search excluded all developments related to tissue and biomedical engineering, electronics, and other industrial non-related sectors. Only studies meeting these criteria were considered for further analysis complemented with those that allow a more in-depth analysis of these studies.

**Figure 1:**
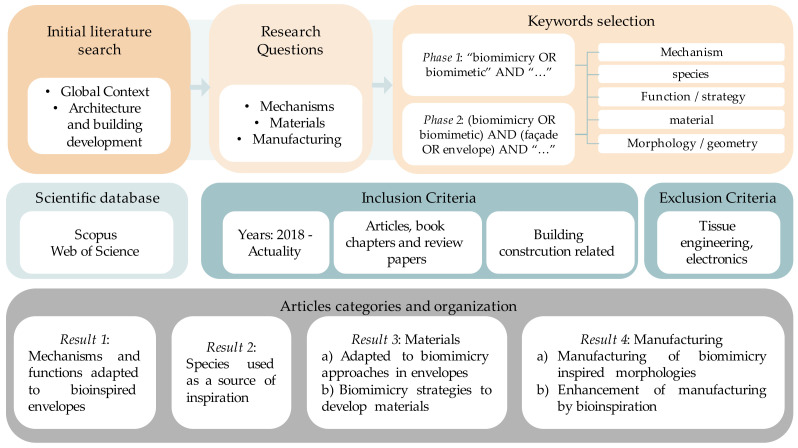
Research methodology proposed for the literature review.

Figure 1 depicts the framework proposed for the literature review. The resulting papers were categorized into four types of results that helped in answering the research questions:

- *Result 1:* Mechanisms and functions adapted to bioinspired envelopes.
- *Result 2:* Species used as a source of inspiration.
- *Result 3:* Materials: (a) adapted to biomimicry approaches in envelopes, and (b) biomimicry strategies to develop materials.
- *Result 4:* Manufacturing: (a) manufacturing of biomimicry-inspired morphologies and (b) enhancement of manufacturing by bioinspiration.

The first two types of results aimed to answer the first question related to the existing responsive building envelopes developed with a bioinspired approach. Here, the search aimed to recollect the role model, meaning the specie or species that served as inspiration, the mimicked features and mechanisms, the application, and the used approach, to understand the physical phenomena and the stimuli that may allow a responsive behavior and to obtain a guide on how to develop such envelopes. The third type of result related to the research question by showing the materials and applications that have allowed or potentially can be adapted to responsive building envelopes. Finally, the fourth type of result allowed us to understand how these materials have been processed to obtain the desired products. In some cases, the same article complied with a different type of result, depending on the main subjects discussed.

In many cases, the studies focused altogether on the selected materials and the manufacturing process. However, in this review, they were divided into two sections to understand how they have been implemented into biomimicry-based solutions and then to explore their role together in the discussion.

## 3. Biomimicry in Responsive Envelopes and Applications

Usually, the common features for a biomimicry-based adaptation for building envelopes are conditions related to heat and temperature, air and wind, light and solar radiation, and water [17,46]. Therefore, understanding these processes in the role model and the effects of those phenomena in the building envelope is usually needed to integrate biomimicry-based approaches. In this matter, living organisms’ dynamic mechanisms and responses can be well-adapted to the architecture, material design, and manufacturing of active envelopes [27]. Nature offers skins, envelopes, and structures that react to environmental stimuli and have been optimized through extensive evolutionary processes to cope with the environment and enhance material, water, and energy use [36,46]. It is necessary to know how the envelope may react or change functionally and organically according to the climate condition to implement biomimicry as a source of inspiration for responsive envelopes [47].

This section reviews the current mechanisms and species that have been previously used to develop biomimicry-based envelopes and how the authors approached their solutions. They have been organized by the stimuli from the environment since this comes of great interest among architects, designers, and engineers when designing responsive building envelopes. Some cases in which the authors have proposed their solution to be potentially used in envelopes have also been included.

### 3.1. Water-Related Mechanisms

Moisture in the building envelope from atmospheric sources such as rain, fog, mist, dew, and others can threaten the integrity and durability of a building envelope, which, if not treated carefully, can lead to damages and degradation due to, for instance, corrosion [48,49,50]. A suitable performance for an envelope must ensure thermal and hygrometric indoor comfort [51]. Moreover, this moisture can be used as a water source, and water harvesting can also be adapted to the building envelope as a source of drinkable water. Therefore, since envelopes represent large surface areas, they can be a source of water harvesting from fog capturing and dew condensation.

Water availability is a critical issue and is considered a significant problem for one-fifth of the world’s population. According to the United Nations Children’s Fund (UNICEF) [52], by 2025, half the world’s population could be living in regions facing water scarcity, and as many as 700 million people could be displaced by 2030 for this same reason. Water can be extracted from the air’s moisture by approximately 1000 m^3^·day^−1^ [53], representing a sustainable strategy for safe, decentralized access to drinkable water [54]. This atmospheric water is equivalent to about 10% of the Earth’s freshwater sources from lakes [55] and 15% of the total surface of available water resources in arid regions [56], representing a base technology nowadays for water collection [54,57,58].

Atmospheric water harvesting (AWH) includes moisture capture, water release, and a filtration or purification process [54], which basically involves two types of technologies: cooling the air below the dew point and an absorption–regeneration technique, which includes the use of absorbent and adsorbents. In this matter, sorbents can capture vapor molecules spontaneously by physical or chemical sorption and then release the captured water upon energy input for collection [53,58], which, depending on the base material, such as hygroscopic salts, polymers, and composite adsorbents, may operate in a broader range of relative humidity [59]. Adsorbent materials, such as silica gel [60,61,62], zeolite [63,64], metal–organic frameworks (MOF) [65,66], and hydrogels [58,67] have been previously applied for water harvesting in soil irrigation, environmental cooling, and drinkable water systems, among other applications.

Dew and fog deposition varies from rainfall deposition since the formers present string variations depending on the nature, extension, and orientation of the surface they came in contact with, compared with the latter, in which it moistens proportionally and with the same amount of water [68]. Fog and dew are the products of the water vapor in the air condensed to form water droplets. These phenomenon can take place on a surface if the surface temperature is equal to or cooler than the surrounding air [69]. Then, this formed drop rests on this surface, forming a contact angle that characterizes the surface wettability. When this contact angle is less than 90°, the surface is considered hydrophilic in nature since it attracts water that clings to the surface [70]. These moisture harvesting mechanisms are found at different levels in animals and plants in any climate.

Biomimetic and biomimicry approaches have demonstrated suitability to adapt to water stimuli for responsive building envelopes. Plant leaves and some invertebrates show the most efficient and developed known systems for water harvesting, in some cases as a coping mechanism to water scarcity in their regular environment and as a means to secure fresh water [71,72]. However, their surface textures tend to be textured and heterogeneous, leading to a wettability behavior that could be described either by a Wenzel state, in which the surface is completely wet, leading to a hydrophilic surface, or by a Cassie–Baxter state, in which the drop sits on the surface, but there is trapped air between the asperities, leading to a hydrophobic surface [69,73,74].

Some well-known examples of species that have inspired biomimetic approaches that could develop water harvest systems recently are: different species of cactus [57,75,76,77,78,79], spiders and spider silks [80], *Triarrhena sacchariflora* [81], pitcher plants [82], such as the *Nepenthes alata* [83], cicadas [74], and beetles [56,79,84,85]. According to Li et al. [83], even if cactus and spider silk have been well-studied, their gradient surfaces can only move the harvested water droplets over a limited drop-sized distance, which leads to a slow speed and limited practical use. Thus, these biological principles can be adapted to design active facades, considering hydrophilic and hydrophobic behavior.

Regarding the design and functionalities of active envelopes, plant leaves may present possible solutions since they have a responsive behavior to heat and moisture, the geometrical and material properties. They are known to influence heat and moisture dissipation due to their morphological traits, such as surface corrugations, textures, trichomes, and sunken stomata [86].

An early work developed by Holstov et al. [87] explored the applicability of wood-based hygromorphic material in a laboratory large-scale external application. Here the authors addressed the challenges in designing and producing hygromorphic composites, including four fabrication methods: gluing, mechanical fixing, spot gluing, and direct lamination. The authors proposed as a matrix a mix of two-part epoxies and polyurethane glues.

In the works of Rupp et al. [86], a breathing skin concept was explored during a one-week intensive interdisciplinary workshop at the cluster Image Knowledge Gestaltung (Berlin). The aim was to target evapotranspiration behavior and shape-change characteristics of leaves to design foldable geometries that can be transferred to large-scale applications related to building architecture. Another approach was developed by Andrade et al. [88] inspired by the *Ammophila arenaria*, which adopted a problem-based approach in three stages: two observation studies; parametric modeling of the leaf movement, using Grasshopper; and experiments with bimetal, a stimuli-responsive material that curls up when heated. The *A. arenaria*, commonly known as marram grass, is usually rolled except under humid environmental conditions [89]. Its sophisticated morphology enables it to adapt well to water and salt stress, triggering a reversible leaf movement [88,90]. The authors identified strategies that can serve as inspiration: the reversible leaf-rolling mechanism, the location of the bulliform cells in the epidermis that determine the closing path of the leaf; the lengthwise cone-leaf closure shape; and the cross-section morphology. Their final approach included the first three, applying the formers in their parametric algorithm. The latter was implemented in the versions of the responsive modules. This strategy was proven to create a shape-changing smart material. The authors suggested that these findings carry a high potential in developing thermal and radiative responsive shading facades.

Later, a problem-based approach based on Carl Hastrich’s iterative spiral and using the hygroadaptive mechanisms of the *Silene Amphorina*, an endemic plant of the Numidian territory, was proposed by Teraa and Bencherif [91]. Here, they proposed to compare the indoor hygrothermal comfort behavior of the Royal Tulip Hotel in northeastern Algeria to a biomimetic scenario. The analysis included a study of the dynamic, sensitive mechanism of the *Silene Amphorina* to air humidity shown by their leaves’ surfaces. The selection for the endemic plant use as a source of inspiration was based on a process nurtured by a questionnaire with scientists and local inhabitants. The transpiration phenomenon is carried out through the stomata of the leaves, which allows a regulation of the opening and closing that responds to the degree of humidity of the environment (relative humidity of the air > 70%). Thus, their study designed a metereosensitive biomimetic envelope and then conducted hygrothermal dynamics simulations using WUFI Plus^®^ software. They found that the biomimetic envelope can regulate the indoor ambient temperature throughout the year and reduce the indoor humidity rate by around 20% in summer, 23% in mid-season, and 35% in winter.

In the case of Rupp and Grubber [92], oak sun leaves were used as a shape inspiration for residential-grade fast-drying shingles. Due to their dissected shape and aerodynamics, oak leaves show fast heat and moisture dissipation. The evaporative and drying processes were analyzed with thermal imaging and weight tracking in warm and cold environments. For the case of a dissected shingle element, when dissipation conditions were favorable, these elements dried faster, providing an effective channeling of liquid water, and the receding of surface moisture was prompted by the lobes and border tips of the leaf-based designs, which were less affected by airflow direction. It also reduced shingle surface area, which can be translated into material savings. The authors provided potential applications as building envelopes, such as graded roofs and leveraged evaporative cooling systems from these shingles that can be paired with a rainwater-storage system.

A theoretical framework for plant-inspired design generation was proposed by Jalali et al. [93] following a solution-based approach. This approach aimed to achieve water harvesting following a four-step plant-to-design path applied to an example of a building envelope panel. In the first step, the authors looked for solutions performed by adaptable plants to water scarcity conditions, choosing water harvesting as their proposed solution. Their selection considered the climate region where the plant is found as a boundary condition, leading them to plants found chiefly in tropical and dry climates. After this, they defined the problem: finding an appropriate response to the water shortage crisis through architecture. In the third step, they pursued the principle of extraction and plant-to-design abstraction, in which they summarized the mechanisms followed by the plant for water harvesting: increasing condensation, reducing transpiration, and facilitating transportation. Finally, they arrived at the principal application in which the conceptual design and architectural design were developed, leading to a water-harvester panel installed in the building envelope that stores the water droplets at the bottom of each panel and with a design that allows the panel to react to climatic conditions of temperature, relative humidity, wind speed, and direction. Their design led to two principles. The first principle was cone-like structures for moisture harvesting, which consisted of cones with a 10° tip angle since they found in the literature that it achieves the highest weight gain of 2 mg·min^−1^·mm^−3^ [75]. The second principle was reducing transpiration by transporting water as fastest as possible. They proposed a Voronoi pattern surface since it is an effective droplet transport mechanism.

A biomimetic Carbon Nanotube Wire (CNW)/PDMS nanocomposite with a superhydrophobic microcolumn surface was developed by Sun et al. [94] inspired by the papillary structure of the lotus leaf surface. This dispositive was tested over several cooling/heating cycles and icing/deicing, demonstrating that electrical heaters, including these biomimetic nanocomposites, show excellent superhydrophobicity and icephobicity. The authors suggested that this kind of dispositive could have potential applications in several sectors.

### 3.2. Heat- and Light-Related Mechanisms

The building envelope solar exposure greatly influences the building’s potential for energy performance [95]. In building facades, controlling parameters such as height, the shape of windows, angles, the shape of shading, and size can be effective in regulating the solar gains of the buildings, as well as influencing the indoor illuminance [96,97], which directly influences the thermal and lighting performance of the building.

Some efforts have been made to use nature as a source of inspiration to deal with solar gains. A self-sustaining, eco-responsive solar house called BAITYKOOL was developed in the framework of the Solar Decathlon Middle East (SDME 2018) [98]. Here, biomimetic solar cells mounted on glass PV panels were integrated into the house architecture. The PV panels’ arrangement was inspired by a sunshine motif acting as a solar envelope. This house was tested for 14 days during the SDME competition, in which an optimized use of solar energy was achieved due to this biomimetic approach, achieving an equivalent of total annual production from their mobile roof, the east, west and south facade of 13,75 MWh, in which 86% was produced due to this biomimetic roof. More recently, Wang et al. [99] developed a transparent and infrared-reflective energy-efficient glass composed of SiO_2_, Al_2_O_3_, and Ag, in which the microscopic structure of the cuticle of the Hercules beetle and the moth eye inspired the physical structure. The unique sponge multilayer structure of the Hercules beetle’s cuticle allows excellent photon management since it provides highly efficient infrared light reflecting. Then, the authors employed the refractive index difference of spongy multilayer structure for infrared reflection. On the other hand, the moth eye consists of a microscopic convex structure allowing an excellent spectral regulation. Thus, it was used for the continuous refractive index change for anti-reflection. According to their results, their biomimetic glass could effectively reduce the energy consumption for cooling by 25.1% in hot weather and for heating by 13.5%, in cold weather.

In the works of Caherpentier et al. [100], an adaptive shading device called Pho’lliage was designed using biomimetics to react to solar heat. In this case, this approach served as a tool for thermal comfort optimization and energy use reduction. The authors used the *nyctinastic* movements as a source of inspiration by including thermal actuation, shape memory, and curved geometry and employing thermobimetal composite metal alloys that react to thermal stimuli. Moreover, Srisuwan [101] was also inspired by the *nastic* movement to develop their facade 3D modeling. The facade was composed of two lightweight triangular plates connected to pneumatic artificial muscles. These muscles controlled the folding and unfolding mechanisms using pressurization. The authors also suggested that this mechanism may be viable for external shading, air ventilation, and light transmission regulation in facades.

In the works of Webb [19], an animal fur perfusion biomimetic-inspired facade, consisting of a fur layer followed by several perfusion facade layers, was modeled in TRNSYS for different climate zones and building typologies. Their numerical results reduced around 50% of the operational energy consumption for all climate zones and building typologies. They identified the source of improving the physical characteristics of the fur lining by providing extra insulation that acted as a barrier to solar radiation, combined with the water-based perfusion inside the facade. Furthermore, the previously cited work of Andrade et al. [88] designed a bi-metal material inspired by the *A. arenaria*, in which they coded a control mechanism for the parametric leaf-blade system in Grasshopper. This mechanism aimed to protect windows from surplus solar radiation during hot days and maximize solar radiation during days with mild temperatures. Grasshopper was also used as a computational tool by Anzaniyan et al. [96]. A simulation was performed with this tool together with EnergyPlus™ of a southern facade of an open office in Tehran. Here, the authors proposed the design, fabrication, and computational simulation of a kinetic facade prototype inspired by the kinetics of *Lupinus Succulentus*, a sun-tracking plant. They aimed to enhance the thermal and visual comfort of the occupants. Their results showed a reduction of about 7% in cooling loads and 48% in electric lighting loads. For Panyaa et al. [102], Grasshopper allowed them to design and find geometric forms of panels that can optimally harness sunlight during the day without disrupting indoor natural lighting. The authors sought inspiration from how leaves react to sunlight in nature. They relied on virtual reality to perform their modeling, allowing them to have an immersive experience while testing their design.

Three cases of the study of biomimetic envelopes are shown in [103]. The first example is the Flectofin^®^, a facade shading system that took as a role model the bird-of-paradise flower (*Strelitzia reginae*). These flowers feature a petal sheath that acts as a perch for birds during pollination; this sheath opens due to the bodyweight force of the bird and exposes the stamens to pollen. The Flectofin^®^ mimics that opening by applying mechanical pressure. To achieve smoothness during the opening and closing, a rib-laminate element consisting of Glass Fiber Reinforced Polymer (GFRP) was developed. Another example is the Flectofold, the aquatic carnivorous waterwheel plant (*Aldrovanda vesiculosa*). This plant feeds itself using millimeter-sized snap traps underwater. These traps consist of two lobes that are connected by a midrib. The facade design transferred this principle into a simplified curved-line folding geometry with distinct flexible hinge-zones, which were optimized by an Italian striped bug (*Graphosoma italicum*) to avoid fatigue in certain areas [104,105]. A third example is the cellular actuator, which is inspired by the plants’ motor cells or bulliform cells of the grass species *Sesleria nitida*. Here, artificial cells consisting of GFPR were made, varying the wall thickness and the cell geometry to obtain different internal pressures.

Wrinkling morphologies found in nature can regulate different physiological, biomechanical, and physical responses. They can be found in planar and curved surfaces in different parts of living beings. Hence, controlling the formation of a wrinkled structure by integrating proper fabrication methods can achieve advances in several fields. Recently, Tan et al. [106] summarized the wrinkling mechanisms, fabrication methods, and further applications of bio-inspired wrinkling patterns. Wrinkles are formed in the skins of some animals. Particular skin morphologies in nature enhance thermal regulation capabilities by using different coping mechanisms [45]. For instance, in most cases, elephants’ environment is characterized by an environmental temperature higher than their body temperature. Temperature changes dictate their behavior during the day. Thus, they have developed several mechanisms to deal with overheating [107]. Elephants’ skin is morphologically adapted to retain water, allowing evaporation as an elephant’s mechanism to overcome overheating. Their wrinkles form a network that enhances their thermoregulation by retaining moisture. Furthermore, these wrinkles may create self-shade regions, reducing heat loads and promoting convection for further heat losses [24,45,107].

Earlier, in the works of Badarnah [24], the author identified that these elephants’ wrinkles might inspire potential building envelopes by applying their evaporation, reflection, and convection mechanisms. Later, it was used as an inspiration for evaporative cooling systems [45]. They aimed to create hexagonal glass fiber-reinforced concrete panels that facilitated cooling. Criteria such as surface roughness and thickness were varied to observe the heat-losing capabilities of these panels.

Similarly, Cheng et al. [108] used a biomimetic wrinkle structure to develop a coating type with a high-performance radiative cooling coating called Bio-RC coating. This large-scale radiative cooling coating comprising high concentrations of BaSO_4_ and SiO_2_ particles was studied in this paper. The design of this material aimed to produce a coating with improved optical properties based on its wrinkled structure and optimized particles, in distribution and size, to enhance the emissivity (achieving 4.5% higher with the wrinkled structure compared to that of the planar coating) and reflectivity, in which the Bio-RC coating with a thickness of ~100 μm can reflect ~95% of solar irradiance. An experimental outdoor test conducted over a year showed a reduction of 6.2 °C in the maximum average indoor air temperature of a building painted with this material. Likewise, the maximum power-saving rate of air-conditioning exceeded 50%.

Some organisms, such as the Antarctic Krill (*Euphausia Superba*), disperse pigments within their skin, originating a rapid and reversible response, actively changing its color [109] due to their sensibility to solar radiation, sensible heat radiation, and latent heat radiation [110,111]. Inspired by this species, an adaptive building interface that uses reversible fluid injections was proposed by Kay et al. [109]. Their study achieved locally adjustable shading and interior solar exposure by injecting and withdrawing pigmented fluids. This approach was based on the hypothesis that intracellular actuation of confined pigment, if used as a material layer at a building scale as a facade, can replicate the biological optical response. Their models showed an improvement in heating, cooling, and lighting energy use by over 30% compared with the electrochromic windows. To achieve this, it was necessary to tackle a dynamic control over multiple fluidic cells that could enable highly localized, digitally programmable shading responses. More recently, Callies et al. [112] used MorphoColor technology to develop a substrate structure whose characteristic feature size is in the same order of magnitude as the wavelength of visible light. The wing scales of the Morpho butterfly inspired this technology. The authors suggested that highly colored solar modules using MorphoColor technology may improve the acceptance of this kind of module on roofs and facades.

### 3.3. Kinetic Mechanisms

Erection mechanisms in nature can also inspire adaptive facades. In the case of Quinn [113], the author depicted their results on the computational methods applied to the design, analysis, and fabrication of two prototypes of bioinspired elastic grid shells by using a pneumatic erection. Three stages were proposed to model the erection dynamics: inflation, beams to support, and deflation. Their process included software such as Grasshopper, Kangaroo, SOFisTik, C#, and Python. Among the challenges to using these techniques, the author mentioned the material selection since they significantly influence mechanical properties such as stiffness and weight, geometry, and fluid pressure during the erection.

Moreover, the biomimetic Research Pavilion 2018 at the campus of the University of Stuttgart was inspired by Diatoms since these algae lock vertically, in which their spines act as flaps preventing horizontal displacement. Here, the elements used are interlocked vertically, bending in one direction, and then locked in a curved non-anchored foundation, leading to a double-curved shell system formation [114].

The ole models found in nature are varied, inspiring different response mechanisms to the environment where the envelope is located. A summary of some selected articles where bioinspired strategies have been applied for developing these envelopes can be observed in Table 1.

## 4. Biomimicry-Based Materials

Recently, biomimicry has been used as a guideline and a source of inspiration to design engineering materials with better mechanical performances, including cellular materials, responsive materials, self-healing materials, self-lubricating materials, self-organized materials, bioinspired polymers, soft materials [2,29,43,44,115], and self-cleaning materials [116]. To achieve this, some approaches look to develop a material that can mimic a specific behavior, e.g., a responsive behavior to a thermal or hygrothermal stimulus. This may be conducted, in some cases, by using materials that already have a certain response and that can be adapted to recreate the mechanism found in nature. In other cases, the response found in nature is a result of the specific structure developed by the own living being. As James Shackelford stated, “structure leads to properties,” meaning that the physical, chemical, and mechanical properties result from the structure at different scales developed by a material, a part, and even a living being over millions of years of evolution.

The development of advanced materials is no stranger to inspiration in nature. For instance, biomineralized materials have inspired typical ceramic synthetization in polygonal grains. The process includes controls of the grains and grains boundaries in such synthetic ceramics, which are analogous to their natural counterpart’s hard building block and soft organic interface. Mimicking this behavior results in preventing failing [31]. Bioinspired and biomimicry-based advanced materials are a current trend in research and development. According to Ahamed et al. [2], materials well-adapted to biomimetic building envelopes may be grouped into those materials that react to external stimuli, functional mimicries for building surfaces, those that seek nature as a source of morphology inspiration for building envelopes, and bio-inspired kinetic envelope systems which mimic bio-movements. Here, materials are classified by their ability to tackle the needs of bioinspired structures and facades and materials that may be enhanced by using bioinspiration.

### 4.1. Hydrophobic Materials

Hydrophobicity in materials and coatings is an attractive property in which biomimicry-based approaches have been actively used lately. Moisture and other sources of water may penetrate and react with the surfaces causing degradation due to corrosion, alkali–aggregate reactions, sealant failure, wind-induced delamination, freezing and thawing, sulfate attack, mold growth, wood decay, loss of thermal resistance of insulation, crack propagation, and water leakage, among others [117,118,119]. Several plants and animals’ skins have served as a source of inspiration to achieve this behavior. For instance, lotus leaves have a hydrophobic surface that repels water droplets, also exhibiting a self-cleaning ability due to having a contact angle > 150°. These properties can be exploited as biomimetic coatings in infrastructures.

Recently, Collins and Safiuddin [117] gathered a collection of materials that have been used as biomimetic coating materials, such as polydimethylsiloxane (PDMS), with a contact angle close to 170°; ultrafine powder coating (UPC), with a contact angle superior to 160°; carbon nanotubes (CNT), nickel (Ni), Ni/Nano-C, and Ni/Nano-Cu, with a contact angle of 155.5°; fluoro-octyl-trichloro-silane-titanium; Janus particles; diamond-like carbon; graphene oxide-silica (GO-SiO_2_); calcium hydroxide [Ca(OH)_2_]; Photopolymer (PP); Acrylic polymer (AP); antimony doped tin oxide/polyurethane (ATO/PU) film; PMMA (Polymethyl methacrylate); PPS/PTFE (Polyphenylene sulfide/polytetrafluoroethylene); copper (Cu); and zinc oxide (ZnO) films. One can find among their properties anti-corrosive behavior, dust-free behavior, anti-abrasion, self-cleaning, anti-fogging and anti-icing, and lubricant and surface coating capabilities. The authors suggested that applications such as buildings, bridges, pavements, and sewers can benefit from these properties. Regarding the buildings, these properties could improve drainage systems, performing as a dust-free, self-cleaning surface on envelopes, preventing the growth of molds and algae, and therefore increasing the service life of buildings. In the study developed by Ghasemlou et al. [120], the authors proposed the use of low-cost raw materials, such as poly(dimethylsiloxane) (PDMS), ternary starch/PHU/CNC (SPC), silica nanoparticles (SNPs), and vinyltriethoxysilane (VTES) to develop artificial robust superhydrophobic surfaces inspired by the lotus leaf (*Nelumbo nucifera)* from Melbourne, Australia. Moreover, Caldas et al. [72] used titanium dioxide nanocoating on a steel welding sheet surface to develop a fog harvesting system for building envelopes. The metal surface was spray painted with a high emissivity white paint (emissivity between 0.989 and 0.992). In this case, the titanium dioxide-based coating performed strong hydrophilicity under ultra-violet (UV) irradiation and strong hydrophobicity in dark conditions.

Silica (SiO_2_) nanofibers and cellulose nanofibers (CNF) were synthesized into a dual fibrous aerogel with a honeycomb-like cellular structure and a nanofiber/nanonet composite cell wall by freeze-drying in [121]. Tetraethoxysilane and poly (vinyl alcohol) were used as starting materials to obtain the SiO_2_ nanofibers.

### 4.2. Self-Healing Materials

The self-healing capacity of living organisms is fascinating and resourceful. It is commonly observed in several cells and tissues, such as bone tissues, skin, and blood [122]. Regarding building envelope applications, this approach has been explored with self-healing concretes. Self-healing concrete is defined by Zhang et al. [123] as “a concrete composite with the ability to repair small cracks automatically, without any external diagnosis or human intervention.” They are of interest in responsive envelopes since they present the ability to adapt and respond to the environment. Additionally, concrete is the most used construction material nowadays; thus, achieving self-healing properties is of great interest [123,124]. These materials can be divided into autogenous healing and autonomous healing. The healing process in the former comes from the material itself, while the latter needs a trigger to activate the process. Autogenous healing is the result of several phases coexisting within the concrete. After the hardening process of the concrete, non-hydrated phases can appear. For instance, a non-hydrated clinker phase (tricalcium silicate, C_3_S, and dicalcium silicate, C_2_S) may react with the water that enters the structure through the cracks and produce calcium-silicate-hydrate. In addition, portlandite (Ca(OH)_2_) can react with the carbon dioxide dissolved in the water, producing some space-filling minerals [122]. According to [124], even if this autogenous healing is useful, the cases studied show limitations and unreliability in the long term.

Moreover, Zhang et al. [123] state that some biomimetic approaches can serve as triggers or mechanisms in autonomous healing, such as electrodeposition technology and embedding shape memory alloy (SMA), capsule, vascular, or bacteria in concrete [124].

Bacteria use or bio-healing is a widely used approach in which the bacteria convert water and some food source, usually added into the concrete matrix, releasing calcium carbonate as a metabolic byproduct and sealing the cracks in the concrete. Even if using bacteria to develop self-healing concrete is not considered a biomimicry-based solution, it is indeed considered in previous works as a bioinspired approach [2,125]. Furthermore, they show the potential to fabricate resilient materials and infrastructures [123].

### 4.3. Biomineralized and Natural Materials

Jia et al. [31] developed an exhaustive review of biomineralized materials that can serve to deeply understand biological hierarchical 3D material architecture as a guide to understanding biological structures. The authors proposed for mineral building blocks 0D granular-shaped, 1D fiber-shaped, 2D tablet/laminated-shaped, and 3D biocontinuous/porous-shaped motifs based on their geometrical features. These kinds of materials exhibit mechanical properties, especially regarding fracture toughness, that make them suitable for biomimicry approaches in architecture [126].

In the study of Sanga et al. [22], clay bricks were developed by mimicking termites’ technique to naturally cemented mound structures. Termites usually feed on cellulose-based materials. To create their shelter, they build above or underneath the ground, changing the soil structure by adding saliva containing mucopolysaccharides which present the cellulase enzyme, converting this cellulose into simple glucose. During this process, and before breaking into glucose, this cellulose is digested into shorter polysaccharides and oligosaccharides. They act as soil stabilizers and binders [127]. Here, the authors used cold-water soluble cassava flour instead of cellulose, and two types of soils were collected from the southern zone of Tanzania. Pure cassava flour, indirectly heated to increase viscosity and cohesion, distilled water, and clay soil were hand mixed and sampled in the form of bricks. The samples were left in the molds for three days and then left to air-dry for twenty-eight days. Their results showed that a cassava paste beyond 6% added to wet soil caused cracking and the development of fungi around the brick surface, but if maintained at 1.6%, a better strength performance can be achieved when compared to traditionally kiln-fired clay bricks.

### 4.4. Composite and Smart Materials

When using composite materials, material testing, design, and fabrications are all considered together since the composite properties, contrary to other raw materials, depend on the nature of their constituents, the proportion, and the fiber’s orientation, as well as the fabrication process. Composite materials such as fiber-reinforced polymers (FRP), glass-fiber polymers (GRP), some metal composites, and concrete composites play a vital role in developing biomimetic envelopes and structures [128]. Thus, material design and fabrication must work together to adapt biomimicry-based approaches to responsive envelopes.

The ITECH Research Demonstrator 2018–19 [47] is one case in which composite materials have been adapted to biomimicry-based envelopes. The kinematic and folding behaviors of the ladybug’s (*Coleoptera coccinellidae*) hindwings were a source of inspiration for developing two compliant elements. Here, an industrial robotic tape-laying process was used to fabricate laminates composed of fiber-reinforced plastic. Composite material properties are highly dependent on the fiber direction. Then, this process allowed them to adjust the fiber orientation and material properties precisely. They were able to process the carbon fiber tapes; however, in the case of the glass-fiber tapes, they had to be processed manually.

Similarly, a smart and adaptive outer facade shading system was developed by Körner et al. [104], inspired by the hinge-less motion of the underwater snap-trap of the carnivorous waterwheel plant (*Aldrovanda vesiculosa*). The design objective was to minimize the bending stresses within the hinge zone. Thus, the materials were tested, focusing on the flexible hinge-zone. A vacuum-assisted process (VAP) was used to fabricate a structure composed of woven glass-fiber fabric, epoxy resin, and polyvinyl chloride (PVC) laminating foil. A material set-up was used to determine the influence of fiber orientation and the implementation of the PVC foil within the composite on the bending stiffness of the hinge-zone. The BUGA fiber pavilion at Heilbronn, Germany [129] is another biomimetic case in which carbon and glass fiber composites were used as the constituent of a composite material. The aim was to construct a bone-like lightweight dome. Core filament winding and robotic fabrication were used as manufacturing techniques to obtain the composite material.

Moreover, these principles applied to fibrous polymeric composites can be applied to other materials. For instance, Allameh et al. [130] developed a nacre-inspired concrete reinforced with chopped fiberglass and chopped carbon fiber and a matrix consisting of masonry cement and graded sand (Quikrete^®^ 1102) to enhance the brittle property exhibited by traditional concretes in construction. The biomimicked composites were achieved by using 3M spray adhesive 80 as a soft polymer. The authors proposed this as a means to endure the effects of earthquakes. This material was possible by using an additive manufacturing technique of concrete deposition, increasing toughness.

The Bouligand structure in the dactyl club of mantis shrimp bioinspired a helicoidal printing pattern in the studies of Liu et al. [131]. The mixture was based on cementitious materials such as general-purpose cement, 30 wt% of ground granulated blast-furnace slag, and 25 wt% of silica fumes. Natural river sand was added as aggregate, and in some specimens, steel fibers were added to make a fiber-reinforced mixture. The existence of these fibers and their orientation changed the results on the specimens regarding energy absorption, peak impact force, impact duration, and porosity. Concrete tiles were developed inspired by animal fur to achieve better insulation performance by Hershcovich et al. [36] and to achieve evaporative cooling mimicking the elephant’s wrinkles by Peeks and Badarnah [45]. The compressive, flexural, and bonding strength of concrete matrices have been widely studied, especially for casted concrete. However, when this material is used to fabricate structures by using additive manufacturing techniques, in some cases, the material stacking is made layer-by-layer, resulting in mechanical anisotropy, void development that weakens interfaces, and a high need for a thorough design process to achieve the desired results.

A material that has gained much interest recently regarding the use of AM in construction is clay. The literature shows that it can be very well-adapted to uses such as non-structural blocks and partitions, components for shades and linings, and non-structural brick vaults [132]. In addition to this, they have gained ground against the common use of concrete since the latter currently presents significant challenges to be compatible with the sustainability and resilience necessary in cities [133]. Various studies have addressed the sustainability of clay [134,135,136] and its capacity to generate resilience against disasters [137,138].

Termite mound soils have been widely used in the past as construction materials for bricks. However, a major challenge with these materials is their limited availability. Thus, Sanga et al. [22] used termite mechanisms to produce their mound soil as a biomimicry-inspired strategy to produce alternative clay bricks. Clay may also be used as a biomimetic coating material. In the case of Mu et al. [139], biomimetic superhydrophobic cobalt blue/clay mineral hybrid pigments were produced, which can act as a self-cleaning, anticorrosive surface. In Dong and Zhang [140] the authors proposed superamphiphobic coating-based nanoclays with fibrous, plate-like, and porous microstructures. They suggested that these developments may help in developing anti-wetting coatings.

Bimetals and metal composite alloys have been adapted in several applications due to their capabilities to respond to thermal stimuli. In the case of [88], the *Ammophila arenaria* leaf-rolling was mimicked by using bimetals that responded to temperature variation and solar radiation, conceiving a future proposal of responsive envelopes. Furthermore, in Charpentier et al. [100], thermobimetals were used to develop an adaptive shading device that mimicked *nyctinastic* kinetics. However, the authors state some challenges regarding the non-uniformity of the temperature-driven deformation of the bimetal.

Interlocking features are often present in bioinspired structures, such as nacre, bones, dermal-epidermal micro-ridges of human skin [108], beetle’s wings [85], the hooks and bulbs of endoparasitic worms, *Pomphorhynchus laevis*, and the alligator gar scales, among others [2,141,142,143]. Interlocking shows mutually dependent interactions between physical objects at their interfaces [143] acting in structures as a toughening mechanism [144]. Therefore, several bioinspired interlocked structures have been developed for different scenarios. They can be classified as static interlocks and regulable interlocks. The former is characterized by enhanced interfacial adhesion, whereas the latter stimulates dynamic responses that lead to multiple functionalities [143]. In this sense, Topologically interlocked materials (TIMs) have recently emerged as a class of architectured materials consisting of stiff building blocks of well-controlled geometries. These structures can slide, rotate, or interlock, collectively providing regulable mechanisms, structural properties, and functionalities [145].

In the works of Srivatsa et al. [141], nacre-bioinspired brick-and-mortar structures of MXene/Polymer nanocomposites were modeled at the microscale using analytical and numerical methods based on finite elements to estimate elastic properties. The design led to an interlocking mechanism between the MXene (Ti_3_C_2_T_x_) fillers in the polymeric matrix (epoxy-resin and polyvinyl alcohol (PVA)). This nacre-bioinspired interlocking promoted an effective load transfer from the polymer to MXenes, and a strength and damage resistance, due to an increase of the weight/volume fraction of MXenes. The authors found that these bioinspired designs increased Young’s modulus by 25.1% and the elastic stress capacity by 42.3%. The authors suggested that nacred-inspired interlocking mechanisms may help control and optimize structure properties.

Moreover, in the case of Mostert and Kruger [146], topological interlocking was employed as a biomimicry principle to facilitate filament bonding during the deposition in concrete 3D printing. The authors altered the interlayer surface with a cross-sectional square, sinusoidal, and zigzag geometric nozzle to achieve topological interlocking. Their results showed that even if the patterns obtained were not fully interlocked, the interlayer bond strength improved at around 66%, 59%, and 29%, respectively, on the selected nozzle geometries.

## 5. Biomimicry-Based Manufacturing Techniques

Many industrial sectors, such as aeronautics and aerospace, automotive, and biomedical, among others, have made significant changes in their manufacturing processes due to the recent developments of industry 4.0, which has led to innovative products with high-quality products, reducing time-to-market, materials, and energy use, with a higher degree of geometric freedom [147,148,149]. These changes include transfer from physical to digital connections, connecting embedded systems and intelligent production processes, using sensing and control tools, augmented reality, cognitive systems, additive manufacturing, advanced materials, autonomous robotics, and digital design, among others [3]. All this has allowed the development of new systems and the inclusion of new technologies, often associated with processes with a more significant impact on sustainability. However, these effects have not been replicated in the same way in the construction sector, which has been slower to adapt to these new and disruptive technologies.

Moreover, the latest development in fabrication and manufacturing techniques have increased active and adaptive facades in buildings. The production capabilities achieved with Industry 4.0 and biomimicry-based approaches have now provided opportunities for innovations and improvements in building facades [19]. However, practical product fabrication depends heavily on geometry, material, and process parameters, which are intrinsically related. Materials play a significant role in reaching the goals of greener and more sustainable buildings, but they must be accompanied by manufacturing and material processes that tackle low demand for resources such as energy, water, and materials [2]. Thus, acknowledging the role of manufacturing in achieving responsive envelopes is key to properly addressing this sector’s sustainability.

### 5.1. Subtractive Manufacturing

Fabrication techniques encompassed as subtractive manufacturing have also been applied as a manufacturing technique to develop systems that may form active envelopes. With the development of computer numerical control (CNC), massive automated production has been possible in several industrial fields [150,151]. Machines, such as CNC millings, allow the fabrication of products with high precision, coupled with robotic arms with multiple joints and degrees of freedom (DOF), nowadays integrating a technology that includes decision-making according to the environment and objects during production [150]. Furthermore, the products from this technique could result in interlocking component geometries, facilitating the assembly of such products and achieving good building energy performance without requiring high-level training [32].

CNC milling has been previously used to produce modular facades, achieving complex geometries and surfaces [152]. Nevertheless, the literature found within the search period regarding using subtractive manufacturing techniques for active or regenerative envelopes is quite limited. The study developed by Peeks and Badarnah [45] that used elephant wrinkles to inspire evaporative cooling applied CNC to create a physical model and then created a mold by using vacuum forming. The concrete panel was cast into the panel and then heated and sprayed with water. Once completely cured, the panels were finished by using CNC-type machines again.

The accuracy obtained by CNC machines may allow further development of active facades by creating high-precision molds. Biomimicry approaches that may be inspired in complex surfaces to enhance the performance of envelopes could achieve a more versatile development with these techniques. Furthermore, the level of precision may achieve surface patterns that may promote the hydrophobic or hydrophilic behavior of plants and animals [69,73,74] or thicken the boundary layer to enhance the insulation of the surfaces [36]. However, as stated in [32], automated CNC processes may produce interlocking parts to help achieve modular prefabricated active envelopes. However, some issues regarding the sustainability of the process and control may still challenge the use of CNC-based manufacturing for developing responsive envelopes.

### 5.2. Additive Manufacturing

According to the ISO/ASTM 52900:2021 [153], additive manufacturing (AM) is a process of joining materials to make parts from 3D model data, usually layer upon layer. AM has several advantages with which complex shapes can be generated, with geometric freedom and a high degree of detail [33,147] and automation [148,154,155], reducing the need for human intervention in the processes, costs, investment times, and risks associated [34]. Therefore, AM can reduce material waste, the labor time of the workers associated with the project, and the time associated with the manufacturing process, improving sustainability in construction [35,156]. AM capabilities of improving sustainability and reducing environmental impact have been widely addressed in the literature, in which automation and digital design show an opportunity to reduce material use and errors, positively impacting the environment [156,157,158,159,160]. Because of this, together with the geometric flexibility offered by this fabrication method, AM can answer some issues and difficulties encountered when designing and implementing active and regenerative envelopes, playing an active role in architecture and future energy-efficient solutions when compared with traditional manufacturing techniques [28,161].

AM is nowadays considered a suitable manufacturing process in the building construction sector. Although, there are still significant challenges surrounding this manufacturing method. A practical AM fabrication depends heavily on geometry, material, and process parameters, and a broad knowledge of how these play their role in AM in the construction sector is still a research trend. Even though some efforts still need to be made, AM and nature have been used together to achieve better results.

For instance, in the works of Felbrich et al. [162], the authors acknowledged the need for architectural envelopes of large sizes. Thus, the authors compared the fused filament fabrication (FFF) manufacturing process to the shell formation on land snails as an opportunity to upscale and adapt FFF, which usually develops small volumetric objects, into a process that can address the need for large-scale flat double-curved objects. They propose a novel rapid additive manufacturing concept based on four biomimetic transfers. The first one consisted of an integrated composite building process. Then, the second biomimetic transfer was based on a continuous six degrees of freedom (DOF) extrusion of laterally attached shell strips. For this, the authors proposed a widened nozzle cross-section and lateral attachment of multiple strip segments to form shell geometry, curved in one direction. Thirdly after that was a post-extrusion modulation, since the snail changes and modulates the soft periostracum after extrusion through muscular movement until it eventually hardens entirely. Here, the authors proposed this biomimetic approach to achieve doubly curved surfaces by adapting the snail strategy to develop a slow and highly accurate controlled cooling process, assisted by a flexible modulation mechanism. The fourth consisted of the possibility extruding bulk material in six DOF. Finally, they proposed periodic building programs as a foundation for computational design. The first approach of this proposal was executed using an industrial robot.

Ceramic stereolithography (CSL) is an additive manufacturing technique that creates 3D ceramics using the layer-by-layer photopolymerization of a ceramic suspension. One of the main features that could make this technique suitable for biomimicry-based approaches is that one can achieve high-accuracy builds and objects [163]. This technique was explored by He et al. [164]. The authors covered material design and characterization, including key process parameters and the effects of the geometry retention of fabricated overhangs, using ceramic stereolithography (CSL). To do that, they proposed CSL-based ceramic fabrication using elasto-viscoplastic ceramic suspension as the feedstock material, a mixture of fine ceramic particles, and a liquid photopolymer resin. Several experimental and analytical studies were performed. The authors acknowledged the potential of this fabrication method in developing biomimetic heat exchangers, among other applications. This technique could potentially be implemented into active facades.

In the case of Liu et al. [131], the authors were inspired by the Bouligand structure in the dactyl club of mantis shrimp, which allowed them to perform a concrete 3D printing following a helicoidal pattern and four pitch angles 0°, 15°, 30°, and 45°. Their results showed that those specimens printed with a helicoidal pattern performed better in terms of impact duration, peak impact force, and energy absorption. Moreover, the authors also observed the effects of reinforcing this mixture with steel fibers, which led to complex cracking mechanisms.

Freeform structures refer to complex geometry buildings and their resisting and non-resisting structural parts [128]. They are desirable in architecture since their curvature may withstand higher lateral forces, and less material is needed when compared with straight walls [28,128,165,166]. To produce a freeform structure, in some cases, such as with composite materials, a molding process is usually needed. The mold surface plays a key role in the quality produced. Here, contact molding, filament winding, and resin infusion require a one-part mold, while press molding, for instance, requires two-part molds [128]. Another manufacturing technique compatible with freeform design in architecture is three-dimensional concrete printing, or 3DCP, which achieves better geometric performance when printing in straight lines compared to curved lines. Even if this technique has not been widely exploited to link freeform structures and biomimicry-inspired architecture at the moment of this review, previous works [128,166,167,168] have shown the ability of this technique while printing curved walls that can be of interest to develop responsive facades.

Several approaches to include biomimicry principles in design and fabrication have been followed regarding AM in polymeric materials and micro and nano-scale fabrication. However, large-scale AM is still in development and faces other challenges regarding the brittle nature of concrete, the most used material in construction. Additive manufacturing in construction includes various technologies such as D-shape and extrusion-based technologies, such as 3DCP and contour crafting. In 3DCP, the material travels from the hopper to the extruder via a concrete pump, then the material is deposited by controlling the movement of the extruder [28]. It has shown great potential in developing a variety of structures and active development in recent years. It offers design freedom for architects and engineers, and it can be well-used if accompanied by some techniques such as topology optimization or bioinspired designs. However, this technology is still in its early stages, resulting in a lack of knowledge, design rules, and guidelines, and an understanding of structural integrity, process, materials, rheology, and mechanical properties [28,169,170]. There is still an active research field related to the development of 3DCP. For instance, when working with this technology, one must deal with the anisotropic behavior derived from the layered structure resulting from the extrusion mechanisms during manufacturing, which contrasts with the isotropic behavior of cast concrete [171,172,173]. However, early studies have shown weaker and more porous, interfacial joints in between filament layer components derived from 3DCP when compared with their cast concrete counterparts [174]. Other aspects that must be considered are the integration of reinforcement during 3DCP, such as fibers and steel cables, into printed filaments or between printed layers, textiles, and others [128,175,176,177].

This 3DCP technology has been used together with biomimicry or biomimetic approaches. For instance, in Suntharalingam et al. [170], a numerical study on the fire performance of a biomimetic 3D printed concrete wall was performed, in which cellular structures demonstrated superior insulation fire rating compared to the rest of the configurations. In the case of [131], it was used to develop bioinspired Bouligand structures, in which the authors found a relationship between the pore size and printing patterns. However, the authors also stated the differences between lab-scale and industrial-scale tests, regarding, for instance, the shear-induced particle migration due to the concrete transportation through a short or long hose, depending on the case.

Topology optimization (TO) can be an answer that links design, biomimicry, and digital form-finding processes [178]. TO is a “mathematical method which spatially optimizes the distribution of material within a defined domain by fulfilling given constraints previously established and minimizing a predefined cost function” [179]. It may allow searching for optimal shapes, some of which can follow hierarchies present in nature while simultaneously decreasing costs and material and energy use [29]. The use of biomimicry in architecture can work together with TO since their principles are similar. In nature, material and energy use tend to look for a minimal use or a “just enough” principle [28]. However, it works well with the capacities of AM and 3DCP [180]. For instance, Sippach et al. [178] developed a lightweight biocomposite-based structure whose morphology was biomimetically inspired by using a unicellular microplankton as a role model. The fabrication was achieved by using tailored fiber placement (TFP), an additive manufacturing technique that allows a flexible orientation of their fibers.

In the same way, the study carried out by Abdallah and Estévez [181] shows a bio-inspired topological design both in the properties of the material (clay) and forms of development that seek the shortest connections between two nodes. This study relied on visual parameters such as the buildability of such structure; however, since it was an early stage for materials such as clay, it lacks measurements of the mechanical performance of the blocks obtained. In the case of Mostert and Kruger [146], the authors used topological interlocking as a biomimicry principle to facilitate concrete filament bonding when depositing the mixture during concrete 3D printing. Their hypothesis was based on the fact that by doing this interlocking, shear stress would appear, enhancing the mechanical resistance. Thus, the authors proposed a change in the nozzle geometry proposing three nozzle patterns: square, sinusoidal, and zigzag. According to their results, process parameters such as nozzle geometry, pattern geometry, printer setup, and print parameters are key to controlling interlocking during extrusion.

Cellular design has shown great interest lately due to the development of AM techniques. They are geometrically based structures in the topological space, which are very frequently found in nature [182]. These can be divided into closed-cell and open-cell typologies. The first case is generally used in AM as a strategy for material reduction, such as the honeycomb structure, which is applied not only in AM but in different techniques and fields to achieve lighter parts with attractive mechanical performance. Closed-cell structures are not fully adapted to their application in all AM techniques. They can occupy space as cubes, prisms, dodecahedrons, blunts, and elongated dodecahedrons [182,183]. Some authors consider these cellular or lattice structures as biomimetic structures since they resemble natural porous materials [126]. They offer the opportunity to provide favorable responses since properties can be modified locally, especially if accompanied by some AM techniques, such as energy absorption, vibration, impact performance, thermal insulation, and sound performance, among many others [28,115,182,183]. Cellular structures can contribute to better use of material and energy when fabricating the envelopes and perform dynamics responses such as hygrothermal responses, airflow balance, and others [184].

### 5.3. Other Manufacturing Techniques

If a surface pattern must be mimicked to enhance properties, micro- and nanofabrication techniques can offer possibilities to achieve these objectives by reproducing specific patterns at different scales. A major challenge of these techniques is that they are still to be a low-cost solution. However, studies using such techniques have been increasing lately [72]. Soft lithography is a fabrication technique that can be used for obtaining microstructures, and it is based on printing and molding using stamps with the pattern of interest [185,186]. This technique can be used to imprint some biological patterns. In the case of Caldas et al. [72], a 3D laser lithography to form a mold and a nanoimprint lithography technique were used in bio-mimicked structures for fog harvesting. These were subsequently used for the fabrication of superhydrophobic coatings.

Moreover, [120] developed a two-step soft-lithography process to transfer the lotus leaf hierarchical patterns onto ternary starch/PHU/CNC (SPC) films. They cast a stirred Poly(dimethylsiloxane) (PDMS) mixed with a curing agent and which was degassed to remove air bubbles into the flat lotus leaf in a Petri dish. Then, they performed a spin-coating procedure to assemble a low-surface-energy thin coating on the poly(dimethylsiloxane) (PDMS) over these microstructure forms by the SPC films. Silica nanoparticles (SNPs) were grafted with vinyltriethoxysilane (VTES) to form functional silica nanoparticles (V-SNPs).

Electrospinning was used during the fabrication process developed by Zhang et al. [187] to obtain a solar-driven lotus-inspired biomimetic evaporator (LBE). Likewise, it was implemented as a sol-gel-based electrospinning process by Dou et al. [121]. The authors developed a semi-template method to fabricate biomimetic-architecture silica/carbon dual-fibrous aerogel. The structure achieved was a honeycomb-like cellular nanofiber/nanonet. The methods were based on freeze-drying the homogeneous dispersion of SiO_2_ nanofibers and cellulose nanofibers co-suspensions. The aim was to provide a thermal insulator for harsh conditions. Their results showed an ultralow thermal conductivity of 0.023 Wm^−1^K^−1^, superior flame retardancy, and excellent structural stability that allowed the aerogel to completely recover under large compression and buckling strain of 80%. However, the authors reported fatigue resistance over 200,000 cycles.

A summary of selected studies in which materials and their fabrication process have been accounted together is depicted in Table 2.

## 6. Discussion

Biomimicry is an innovation inspired by nature [23]. Many researchers have been looking for inspiration in nature to improve system efficiency and reduce the environmental impact of products or systems in the face of global challenges such as climate change and population growth, which involve increased demand for resources and services. For this reason, some researchers became further interested in sustainable systems or clean life cycle products by framing biomimicry principles, which are an opportunity for the adaptation of the construction sector in its transition to more sustainable methods and application of technologies in planning, materials, execution, and operation. Obtaining a responsive envelope is a possibility to accomplish energy savings and have a low environmental impact due to their role in regulating buildings’ internal and external exchanges. Although many efforts have been made toward exploiting natural principles as a tool to tackle responsive behavior in envelopes, responsive biomimicry-based envelopes intel a full clean life cycle from their designing, material manufacturing [2], and envelope construction, ensuring envelope sustainability and regeneration, as well as the safety and comfort of occupants, demanding as little as possible from the building’s surroundings (ecosystem). However, the latter ensures the sustainability (or taking care of) of the place where the building is located.

In this context, the literature review focused on answering three proposed research questions aimed at comprehending these previously mentioned approaches.

- What have been the biomimicry solutions previously adapted to responsive envelopes or related and their mechanisms?

Section 3 depicts several initiatives and envelope structures using biomimicry, bioinspired, and biomimetic approaches, which include a wide variety of animal and vegetal species that serve as role models. Responsive building envelopes react to different environmental stimuli seeking to achieve better building performance and, in most cases, improve their sustainable operation. Thus, a deep analysis of these studies gives the foundation to framework the design of sustainable, responsive building envelopes, allowing an understanding of how it was previously performed, but more importantly, how it may be reproduced and enhanced in the future.

These studies have been divided by considering the environmental stimuli that trigger the dynamic envelope mechanism. Nature offers excellent knowledge on adapting skins and surfaces to the environment since most living beings must adapt and cope with several conditions. Thus, among the studies, some examples found inspiration in nature to develop facades and structures that responded to environmental light, heat, and water stimuli. These studies tackle role models, biological functions and strategies, morphologies, materials, and manufacturing techniques, including theoretical, numerical, and experimental approaches.

Plants and invertebrates were the more mimicked organisms currently used to achieve responsive facades. In the last five years, most of the efforts have been directed at developing biomimetic envelopes that include mechanisms that react to light and solar heat and atmospheric water harvesting. At large-scale applications, most efforts sought to adapt the morphology and kinetic mechanisms of the role model into a biomimetic solution. At the micro- and nanoscale, natural inspiration generally sought material fabrication and modification to achieve specific properties, e.g., for coatings applications.

Even if several species have already been used as a source of inspiration, most of the studies analyzed used similar role models and mechanisms. The diversity of the species’ coping mechanisms that one can find in nature, other than those found in this search, gives rise to the need for deeper analysis of how to achieve strategies that reach biomimicry-based envelopes, meaning that there is still a need to make more substantial efforts in integrating biomimicry in architecture.

- How have materials been previously designed or adapted to comply with biomimicry solutions adapted to responsive envelopes?

Natural and synthetic materials have been used to tackle biomimetic, bioinspired, and biomimicry approaches in responsive envelopes. The literature addresses which materials are ‘keener’ to be adapted to biomimicry-based approaches in architecture, as can be analyzed. The literature revealed that some materials had been adapted to a specific morphology that allows a responsive behavior, while others are responsive themselves to specific stimuli and used accordingly when following biomimicry-based or biomimetic-based approaches.

If the approach is based on achieving the role model morphology to sustain a proper environmental response, then the main issue is to achieve the geometric target of such morphology. Traditional materials, e.g., polymers, wood, ceramics, metals, composites, and others, as well as responsive materials, have previously been used indistinctly. Here, one major challenge is the manufacturing process that allows those materials to reach the desired form. The material used for any application and its manufacture interact dynamically since they are inherently dependent on each other, leading to visible limitations when trying to improve the performance of a technology, system, or product. Materials come with specific properties that limit their manufacturing capabilities by using a particular technique, such as workability, machinability, and rheology, among others. These properties are considered as material parameters during the design and manufacturing processes, playing a major role in the final outcome [188]. Even in other applications, the performance depending on the material parameter is still under study, and for newer technologies, such as AM or smart materials and composite, there is still continued development. Therefore, even if the studies in the literature reveal the manufacturing technique and process to obtain the desired envelope, there is still plenty of room for improvement and, furthermore, to control and optimize these parameters to obtain better products.

Traditional and responsive materials have been reported when mimicking the role model’s dynamic mechanism. However, there is an increased interest in responsive materials such as soft materials, self-cleaning, hydrophobic and hydrophilic materials, thermo bi-metal, and shape-memory materials, among many others, since they may adapt easily to the specific response, even without the need to adapt to a particular morphology.

Moreover, the literature shows that not only have materials been adapted to biomimicry, bioinspired, and biomimetic envelopes, but in some cases, these approaches have inspired a new type of responsive material that may achieve better results if included in building envelopes. Material science and fabrication is an active field in many industries, and for responsive building envelopes, it may represent an opportunity to develop further sustainable responses for buildings.

- Which manufacturing techniques have been used to comply with biomimicry solutions, or how has biomimicry enhanced manufacturing techniques adapted to responsive envelopes?

When designing responsive envelopes, manufacturing, material development, use, and applications must be made together. However, manufacturing is still challenging when carrying out a biomimicry-based approach to building envelopes. Nature grows structures, which are done hierarchically. Human-made structures are fabricated, making them highly dependable and limited by the process, geometric, and material parameters.

Traditional manufacturing, fabrication, and construction have long been used to produce our buildings’ envelopes, which has profoundly impacted their forms, geometry, and static behavior. Recent efforts to transition to responsive envelopes may struggle to find large-scale manufacturing processes that appropriately address the envelope’s expected responsive mechanisms. The analysis in Section 5 shows traditional manufacturing processes such as casting, molding, and forming, which allow designers, architects, and engineers to reach the desired role-model-inspired morphology, function, or mechanism. However, there is an increasing interest in using AM due to its virtual geometric freedom, which may comply with the complex nature-inspired morphologies in envelopes. Although, there is still a long way to go in developing this technology, not only because of the limitation of the process, such as large-scale use, materials, and deep understanding of the physical controlling mechanisms, but also the digital barrier that the construction sector still has. Subtractive manufacturing and integrating CNC technologies may also answer the need for a responsive facade, but rigorous process control is necessary to achieve sustainability since waste management is still a major issue when applying these techniques.

Despite all these challenges, the literature has shown that CAD, combined with parametric design, topology optimization, CNC, machine learning techniques, and human–robot interaction, among many others under Industry 4.0, set new trends in manufacturing that may change how envelopes are conceived from the early stages.

However, most of the studies found are biomimetic-based rather than biomimicry-based. There are significant differences between these two concepts [189]. For instance, some responsive envelopes follow the three mechanisms highlighted in Section 3, where most of them draw the principles of plants and animals as they have effective behavioral processes and skin morphologies focused on the solution of a particular issue, e.g., water, heat, and light harvesting, but studying the pinnacles’ underlying mechanisms alone falls into the biomimetic approach definition. Such an approach focused on improving efficiency via technology and innovation to satisfy human needs and adaptation to climate conditions [189]. Moreover, most of these studies tackled manufacturing as a process, revealing the procedure followed to fabricate the material or structure. However, there is still a lack of studies showing how the manufacturing process, material selection, and geometry parameters may impact the final performance of the building. These effects have usually been tackled as a single study in which the material and manufacturing techniques have been analyzed regarding the effects of such parameters [161,166,188].

Furthermore, such active envelopes need particular material characteristics to achieve the pinnacles’ underlying mechanisms. To cope with such mechanisms, natural, composite, or smart materials are needed allowing a dynamic interaction/reaction with the building surroundings for the abovementioned harvesting; for example, they should be capable of self-cleaning or protecting itself from humidity, e.g., with a hydrophobic feature. Having an envelope able to harvest occupant needs as well as self-caring [123] reduces the need to exploit or negatively impact the ecosystem’s services, i.e., water supply, energy provision, air purification, habitat provision, and climate regulation [190].

**Figure 2:**
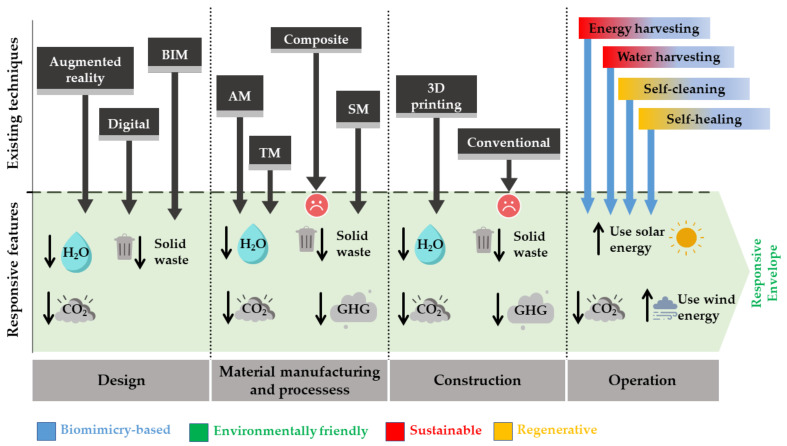
A framework to present current research regarding techniques, materials, and manufacturing techniques towards achieving responsive envelopes, highlighting the bridge (dashed horizontal line) and research gaps (sad face). Arrow directions refer to: down (decrease) and up (increase).

In this context, materials capable of such functions may require complicated and resource-efficient manufacturing techniques, especially when the whole facade is made from these materials rather than only as a covering or coating [108,117,140]. However, few manufacturing techniques are environmentally responsive or even sustainable (based on its three pillars). Among them are the previously mentioned SM and AM. Figure 2 presents a framework of current techniques regarding materials and manufacturing highlighting the bridge and research gaps, towards achieving responsive envelopes. 

The envelope construction process also needs to be considered from the biomimicry perspective, where additive manufacturing helps reduce material waste [39,147]. Various studies highlighted the environmentally responsive features of both techniques, but manufacturing the active envelope’s materials requires rather complex deposition procedures. Yet AM struggles to manufacture a fully responsive envelope. The latter presents a gap in current developments where most responsive envelopes implement materials still not covered by a responsive manufacturing process such as AM. Such materials are often composites allowing the dynamic response of the envelope, while AM is often used for polymers, metals, and some ceramics; instead, traditional manufacturing techniques are often employed for active envelopes. AM as a manufacturing technique for large composites is still in development, representing an opportunity for further studies.

Furthermore, biomimicry implies the measure of success regarding the sustainability of the product or system. The literature offers quantitative indicators of the amount of water saved and recycled water for the envelope construction process. However, greater efforts need to be made in the construction technologies to achieve adaptive and regenerative buildings in the face of the challenges of energy demand, comfort, and decarbonization of the industry. Responsive envelopes are an opportunity, as well as the biomimicry approach, to address its challenges through structural emulation and functional dynamics based on nature.

## 7. Conclusions

Since nature is an inspirational basis for achieving sustainability and requirements for a responsive building envelope, building envelopes present a great opportunity for energy savings, low environmental impact, comfort, and even self-healing. For this reason, a literature review was carried out, from 2018 to the present, on the biomimetic approaches to building envelopes in a global context of the field as well as of architecture and building development, addressing three questions focused on mechanisms, materials, and manufacture:

1. In mechanisms and functions adopted for bio-inspired envelopes, species are used as a source of inspiration.
2. In materials adapted to biomimetic approaches in envelopes and biomimetic strategies for their development.
3. In manufacturing, morphologies are inspired by biomimicry and enhanced through bio-inspiration.

Water availability is one of the most critical issues for the future, so in the findings of water-related mechanisms, the ability of buildings’ envelopes to extract water from the moisture in the air has been remarkable. This atmospheric uptake is found in various animals and plants, which is efficient from plants to spiders because of hydrophilic and hydrophobic behavior, geometry, and material properties. For energy performance, there have been bio-inspired efforts to deal with solar gains with roofs, glass, and adaptive shading devices that respond to solar heat through multi-layered structures such as the Hercules beetle’s cuticle and nyctinasty movements. Additionally, plant-inspired facades for thermal improvement and visual comfort or mechanical pressure-based shading systems and wrinkle morphologies can help buildings’ envelopes, such as the elephants, where evaporation, reflection, and convection mechanisms are applied. Their wrinkles have the adaptability to retain water and have thermoregulation.

The results highlight that the characteristics that make an envelope responsive are achievable with complex materials. These materials require manufacturing processes with conventional techniques capable of creating composite materials. The manufacturing techniques that are able to create these materials are not very environmentally friendly. On the contrary, it was found that AM and controlled SM processes are the most sustainable, but they still need to achieve composite and smart materials of such characteristics. Thus, it is concluded that there is a significant gap in the manufacturing techniques to achieve these materials.

## Figures and Tables

**Table 1:** Selected cases of biomimicry-based envelopes.

Role Model	Mimicked Features and Mechanisms	Application	Approach	Schematic Representation	Ref
Marram grass *(Ammophila arenaria)*	Reversible leaf-rolling mechanism Closing path of the bulliform cells Lengthwise cone-leaf closure shape Cross-section morphology	Shape-changing bi-metal material with potential for thermal and radiative responsive shading facades	Numerical and experimental	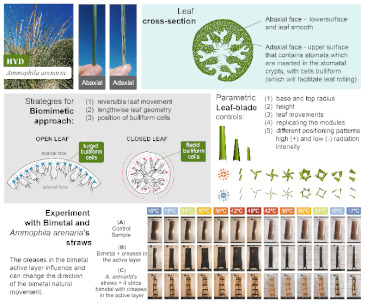	[90]
*Silene Amphorina*	Transpiration phenomenon through the plant’s leaf stomata, focusing on the opening and closing mechanisms as a response to the degree of the environmental humidity	Metereosensitive biomimetic envelope to enhance the indoor hygrothermal comfort behavior of the Royal Tulip Hotel in northeastern Algeria	Numerical	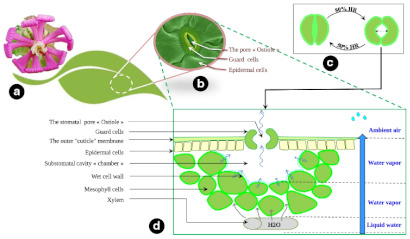	[93]
Oak sun leaves	Dissected shape to achieve faster heat and moisture dissipation Aerodynamical behavior due to the leaves’ lobes and border tips	Residential-grade fast-drying shingles for graded roofing and leveraged evaporative cooling	Experimental	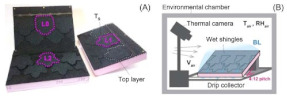	[94]
Cactus, *Eremopyrum orientale*, and *Salsola crassa*	Cone-like structures for moisture harvesting Effective droplet transport mechanism of a Voronoi pattern surface	Water harvesting system for a building envelope panel	Theoretical	-	[95]
Lotus leaves	Papillary structure of the lotus leaf surface	Biomimetic nanocomposite film with a superhydrophobic surface	Experimental	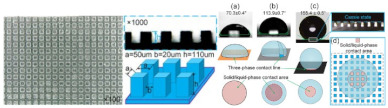	[96]
Solar behavior	Sunshine motif acting as a solar envelope	Biomimetic solar cells mounted on glass PV panels for a self-sustaining, eco-responsive solar house (BAITYKOOL)	Theoretical and Experimental	-	[100]
Hercules beetle’s cuticle Moth eye	The spongy multilayer structure of the Hercules beetle to improve the infrared reflectivity The microscopic convex structure of the moth eye to reduce the visible light reflectivity	Biomimetic energy-efficient glass	Numerical	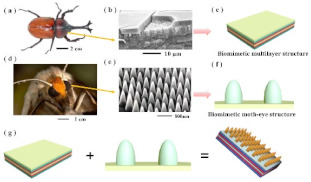	[101]
Animal fur and blood perfusion	Insulating mechanism of animal fur	Fur-inspired building envelope	Numerical	-	[19]
*Lupinus Succulentus*	Sun-tracking kinetic mechanism	Kinetic facade prototype to enhance thermal and visual comfort of the occupants	Numerical	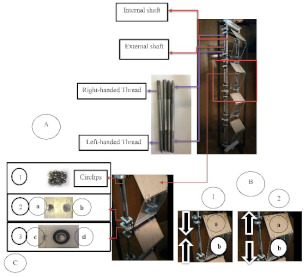	[98]
Leaves	Sun-tracking mechanisms of leaves	Panels that optimally harness sunlight during the day	Virtual reality	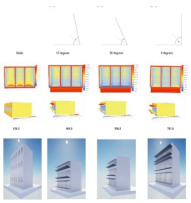	[104]
Bird-of-paradise flower (*Strelitzia reginae*)	The perch-like mechanisms of the petal sheath that activates during pollination by applying mechanical pressure	Facade shading system (Flectofin^®^)	Experimental	-	[105]
Aquatic carnivorous waterwheel plant (*Aldrovanda vesiculosa*) Italian striped bug (*Graphosoma italicum*)	The millimeter-sized snap traps of the *Aldrovanda vesiculosa* used for feeding The flexible hinge zones of the *Graphosoma italicum* structure	Facade shading system (Flectofold)	Experimental		
Elephants’ wrinkles	Evaporative cooling mechanisms of the elephants’ wrinkles by mimicking texture, depth, and morphology	Textured facade panel	Experimental	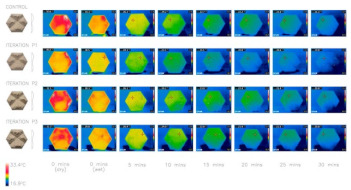	[49]
Humans’ wrinkles	Wrinkled surface to enhance optical features	Biomimetic radiative cooling coating material (Bio-RC coating)	Experimental	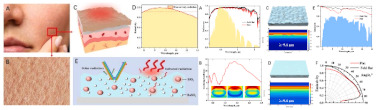	[110]
Antarctic krill (*E. superba*)	The pigment modulation of the Antarctic krill (*E. superba*) that reacts to sunlight intensity	Active building facade using reversible fluid injections to control optical transmission	Experimental and numerical	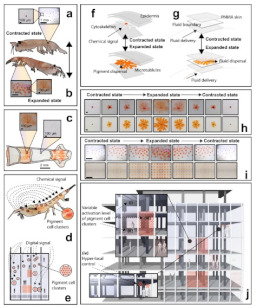	[111]

**Table 2:** Summary of the cases in which manufacturing and materials are addressed together.

Material	Reactive Response/Property	Role Model	Manufacturing/Fabrication Process	Ref
Polydimethylsiloxane (PDMS)	Superhydrophobicity	Nelumbo Nucifera lotus leaves	soft-imprinting lithography	[1,2]
Silica nanoparticles (SNPs)	Thermal insulator	Natural honeycomb	Sol-gel-based electrospinning Spin-coating Roller coating	[1,3,4]
Concrete	Self-healing	Bacteria	Incorporating suitable healing materials to concrete and then using conventional processes	[5,6]
	Enhanced brittle properties and toughness	Nacre	Additive manufacturing	[7]
	Better printing patterns Increased energy absorption, peak impact force, impact duration, and porosity	Bouligand structure in the dactyl club of mantis shrimp	Additive manufacturing	[8]
	Insulation	Elephant’s wrinkles	CNC mold fabrication Vacuum molding Concrete casting	[9]
	Enhanced mechanical resistance	Topological interlocking in nature	Additive manufacturing	[10]
Clay	Enhanced resistance to cracking	Physiology	Additive manufacturing	[11]
	Variable increases in strength depending on the amount of cellulose added to the mix	Termites	Molding	[12]
	Superhydrophobicity	Lotus leaf	Mix preparation under magnetic stirring	[13]

## Data Availability

Not applicable.
